# Riociguat for CTEPH: A retrospective cohort study from a single reference center in Brazil

**DOI:** 10.1016/j.clinsp.2025.100769

**Published:** 2025-09-11

**Authors:** Caio Julio Cesar dos Santos Fernandes, William Salibe-Filho, José Leônidas Alves-Jr, Carlos Vianna Poyares Jardim, Túlio Martins Vieira, Mario Terra-Filho, Rogerio de Souza

**Affiliations:** Instituto do Coração, Hospital das Clínicas da Faculdade de Medicina da Universidade de São Paulo (HCFMUSP), São Paulo, SP, Brazil

**Keywords:** CTEPH, Riociguat, Hemodynamics, Functional capacity

## Abstract

**Objective:**

To evaluate the effects of Riociguat treatment in patients with Chronic Thromboembolic Pulmonary Hypertension (CTEPH) at a Brazilian reference center.

**Methods:**

This observational study was conducted at INCOR-SP and included 8 patients diagnosed with CTEPH between 2016 and 2024 who were treated with Riociguat. Clinical parameters (NYHA functional class, BNP levels, and 6-minute walk distance) and hemodynamic measurements (right atrial pressure, cardiac index, and pulmonary vascular resistance) were assessed pre- and post-treatment, along with risk stratification using COMPERA 2.0.

**Results:**

The retrospective cohort (mean age 56 ± 17 years; 100 % female) comprised 2 inoperable patients and 6 with residual PH post-PEA. Significant improvements were observed in functional class (*p* < 0.05), 6-minute walk distance (increased from 398.5 ± 45 m to 413 ± 110 m; *p* < 0.05), and BNP levels (decreased from 140 ± 191 pg/mL to 28 ± 235 pg/mL; *p* < 0.05). Additionally, risk stratification according to COMPERA 2.0 improved significantly. However, no significant changes were detected in the hemodynamic parameters (mean pulmonary arterial pressure, right atrial pressure, cardiac index, or pulmonary vascular resistance).

**Conclusion:**

Riociguat treatment significantly enhanced functional capacity, reduced BNP levels, and improved risk stratification in CTEPH patients, corroborating international evidence. The absence of significant hemodynamic changes underscores the need for larger, prospective studies to fully elucidate the cardiovascular impact of Riociguat. Importantly, the limited availability of Riociguat in Brazil remains a critical challenge that hinders the effective management of CTEPH.

## Highlights

• CTEPH treatment is challenging and multimodal; only Riociguat is approved.
• Access to Riociguat in Brazil is difficult; limited data exist for local patients.
• The cohort of CTEPH patients treated with Riociguat showed improved NYHA class.
• BNP levels significantly dropped after treatment with Riociguat.
• 6MWD increased, confirming the positive impact of Riociguat therapy.

## Introduction

Pulmonary Hypertension (PH) is a complex condition associated with significant morbidity and mortality. Among its forms, Chronic Thromboembolic Pulmonary Hypertension (CTEPH) is unique in being potentially treatable through surgical (Pulmonary Endarterectomy, PEA), Balloon Pulmonary Angioplasty (BPA), or pharmacological interventions. Riociguat, a soluble Guanylate Cyclase stimulator (sGC), is the only approved medical treatment for CTEPH patients who are inoperable or have residual pH post-endarterectomy.

In the pivotal CHEST-1 trial,^1^ a phase 3, multicenter, randomized, double-blind, placebo-controlled study, 261 patients with inoperable CTEPH or residual pH post-PEA were randomized to Riociguat or placebo for 16-weeks. Riociguat significantly improved the Six-Minute Walk Distance (6MWD) by a mean of 39 m compared to a decrease of 6 m in the placebo group (least-squares mean difference, 46 m; 95 % CI 25 to 67; *p* < 0.001). Pulmonary Vascular Resistance (PVR) decreased by 226 dyn/s/cm^-5^ in the Riociguat group, while it increased by 23 dyn/s/cm^-5^ in the placebo group (*p* < 0.001). Riociguat also improved NT-proBNP levels (*p* < 0.001) and WHO functional class (*p* = 0.003). Long-term benefits of Riociguat were confirmed in the CHEST-2 extension study,^2^ with sustained improvements in 6MWD and WHO functional class for up to one year.

In time, Riociguat became the most used drug for clinical treatment of CTEPH patients. The recently published Worldwide CTEPH Registry,^3^ which evaluated 1009 newly diagnosed CTEPH patients across 34 centers in 20 countries, reported that 64 % of patients were receiving Riociguat one year after revascularization (PEA or BPA) or during follow-up. For BPA patients alone, this proportion increased to 79 %.

Despite global experience with Riociguat, its utilization in Brazil remains limited. Access to Riociguat continues to be challenging in the country, with significant barriers related to medication approval, funding, and healthcare system navigation. Spilimbergo et al.^4^ described the largest Brazilian cohort, comprising 31 PH patients treated with riociguat for three years, including 12 with CTEPH. Improvements in NYHA functional class and a significant increase in the 6MWD (from 394 ± 91 m to 458 ± 100 m; *p* = 0.014) were observed, with a three-year survival rate of 96.7 %. However, detailed hemodynamic and CTEPH-specific data were lacking.

This study presents the experience of INCOR, a Brazilian PH reference center, with Riociguat for CTEPH treatment. This real-world analysis aims to complement international trials by providing local data, critical to understanding the Brazilian patient population and therapeutic access landscape. Clinical parameters (functional class, BNP levels, and 6MWD) and hemodynamic measurements (Right Atrial Pressure [RAP], Cardiac Index [CI], and PVR), along with COMPERA 2.0 risk stratification,^5^ were evaluated pre- and post-Riociguat therapy.

## Methods

This retrospective observational study included patients diagnosed with CTEPH treated with Riociguat at INCOR between 2016 and 2024. Inclusion criteria were confirmed CTEPH diagnosis, Riociguat therapy, and available pre- and post-treatment clinical and hemodynamic data. Variables collected included:

• Demographics (age, gender);
• NYHA functional class;
• BNP levels;
• Six-minute Walk test distance (6MWD);
• Hemodynamic parameters from right heart catheterization (RAP, CI, PVR);
• COMPERA 2.0 risk status.

The median timeframe between pre- and post-treatment assessments was at least 6-months. Statistical analyses were performed with Bonferroni correction to adjust for multiple comparisons. Bonferroni correction was applied only to the primary outcome comparisons (functional class, 6MWD, BNP, and COMPERA 2.0).

This study was approved by the Research Ethics Committee of the Instituto do Coração, Hospital das Clínicas da Faculdade de Medicina da Universidade de São Paulo (approval n° 7.094.336; CAAE: 82,895,024.3.0000.0068). Its conception and reported were in accordance with the STROBE Statement guidelines for observational studies. Waiver of informed consent was granted due to the retrospective nature of the study and use of anonymized data.

## Results

The cohort included all patients with complete pre- and post-treatment data within the study period, a total of 8 CTEPH patients. Two patients were considered inoperable, and six had residual PH post-PEA. The mean age was 56±17 years, and all patients were female. Baseline characteristics are shown in Table 1.

**Table 1:** Baseline clinical and hemodynamic data.

**Parameter**	**Value**
NYHA Functional Class (1 / 2 / 3 / 4)	0 / 1 / 7 / 0
6MWD (m)	398.5 ± 45
BNP (pg/mL)	140 ± 191
COMPERA 2.0 (3-strata: Low / Intermediate / High)	0 / 6 /2
COMPERA 2.0 (4-strata: Low / Int.-Low / Int.-High / High)	0 / 6 / 2 / 0
Mean Pulmonary Arterial Pressure (mmHg)	47 ± 11
RAP (mmHg)	15 ± 6
Cardiac Index (L/min/m^2^)	2.4 ± 0.76
PVR (Woods units)	9 ± 5.87
Baseline therapy	
None	2
ERA	6
Prostacyclin analog	1

6MWD, Six-Minute Walk Distance; BNP, Brain Natriuretic Peptide; RAP, Right Atrial Pressure; CI, Cardiac Index; PVR, Pulmonary Vascular Resistance; ERA, Endothelin Receptor Antagonist.

**Fig. 1:**
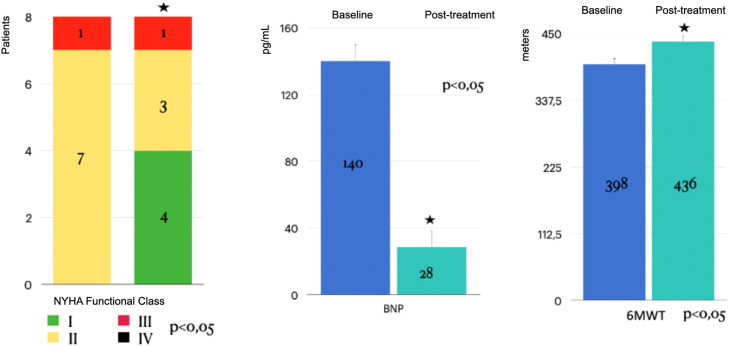
Effect of Riociguat treatment on the NYHA (New York Heart Association) Functional Class, BNP (Brain Natriuretic Peptide) and 6MWT (Six-Minute Walk Distance) in CTEPH (Chronic Thromboembolic Pulmonary Hypertension) patients.

**Fig. 2:**
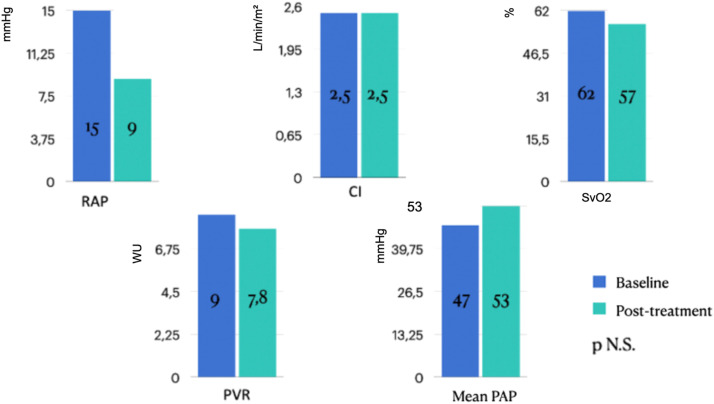
Effect of Riociguat treatment on the Right Atrial Pressure (RAP) Cardiac Index (CI), Central Venous O_2_ Saturation (SvO_2_), Pulmonary Vascular Resistance (PVR) and mean PAP, of CTEPH patients. No statistically significant changes observed (*p* > 0.05). mmHg, millimeters of mercury, WU, Woods Units.

**Fig. 3:**
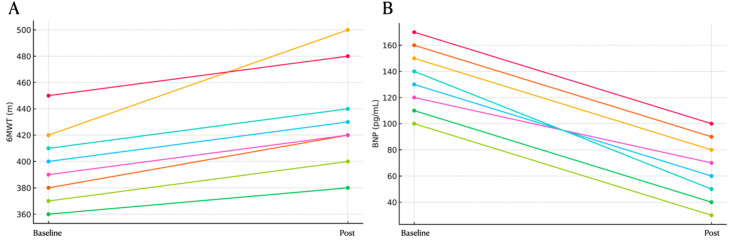
Individual patient trajectories in 6MWD (Six-Minute Walk Distance) (A) and BNP (Brain Natriuretic Peptide) (B).

**Fig. 4:**
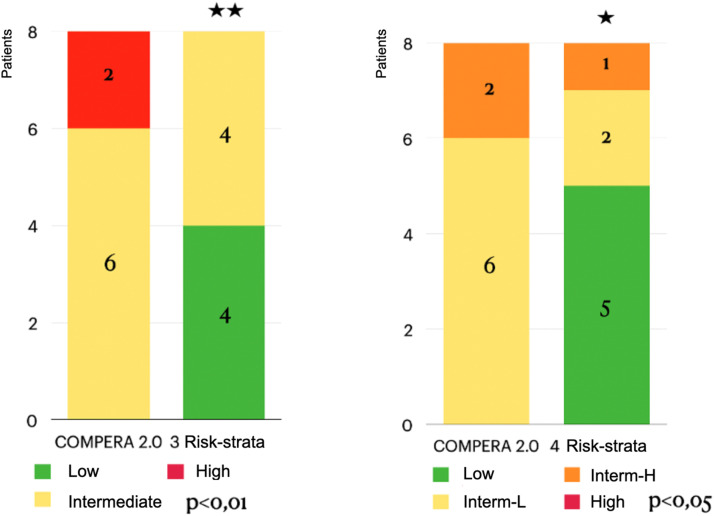
Effect of Riociguat treatment on the COMPERA 2.0 risk stratification of 3 and 4 risk strata of CTEPH patients. Interm-L, Intermediate-Low; Interm-H, Intermediate-High.

Significant improvements were observed in NYHA functional class, 6MWD, and BNP levels even after adjustment (corrected p-values < 0.001). Additionally, individual patient trajectories for 6MWD, BNP, and NYHA functional class were analyzed to assess response variability. Post-Bonferroni correction, significant improvements remained in NYHA functional class (corrected *p* = 0.00009), 6MWD (corrected *p* = 0.00009), and BNP levels (corrected *p* = 0.000003). Hemodynamic parameters remained non-significant, which the authors further discuss below (Table 2, Fig. 1, Fig. 2, Fig. 3, Fig. 4).

**Table 2:** Comparison of baseline and post-treatment parameters.

**Parameter**	**Baseline**	**Post-Treatment**	**p-value**
NYHA Functional Class (1 / 2 / 3 / 4)	0 / 1 / 7 / 0	4 / 3/ 1 / 0	<0.05
6MWD (m)	398.5 ± 45	413 ± 110	<0.05
BNP (pg/mL)	140 ± 191	28 ± 235	<0.05
COMPERA 2.0 (3-strata: Low / Intermediate / High)	0 / 6 / 2	4 / 4 / 0	<0.01
COMPERA 2.0 (4-strata: Low / Int.-Low / Int.-High / High)	0 / 6 / 2 / 0	5 / 2 / 1 / 0	<0.05
Mean Pulmonary Arterial Pressure (mmHg)	47 ± 11	44 ± 12	NS
RAP (mmHg)	15 ± 6	14 ± 5	NS
Cardiac Index (L/min/m^2^)	2.4 ± 0.76	2.5 ± 0.8	NS
PVR (Woods units)	9 ± 5.87	8.8 ± 5.6	NS

6MWD, Six-Minute Walk Distance; BNP, Brain Natriuretic Peptide; RAP, Right Atrial Pressure; CI, Cardiac Index; PVR, Pulmonary Vascular Resistance; NS, Not Significant (*p* > 0.05). Due to data skewness, BNP, Post-treatment is also reported as median [IQR]: 15 [10–30] pg/L.

## Discussion

This study provides a relevant contribution to the understanding of the efficacy of Riociguat in CTEPH by presenting detailed national data, including complete hemodynamic parameters. By demonstrating significant improvements in functional class, BNP levels, and distance walked in the six-minute walk test, the cohort highlights the positive clinical impact of Riociguat in a real-world setting in Brazil. The inclusion of pre- and post-treatment hemodynamic data lends additional robustness to the findings, considering that previous national registries had not explored this level of detail.^4^ These results complement international evidence[3] by providing specific support on the Brazilian reality, contributing to a better understanding of the efficacy and barriers to access to treatment in the country.

Although Riociguat is the only approved pharmacological therapy for CTEPH, off-label use of Pulmonary Arterial Hypertension (PAH)-specific drugs is frequent in clinical practice, especially in resource-limited settings.^6^ Future prospective studies should compare Riociguat to BPA and off-label PAH therapies to evaluate relative effectiveness.

Additionally, PEA remains the preferred curative treatment for operable patients, while BPA has emerged as a valuable option for those with residual pH after surgery or inoperable disease.^7^ The integration of these modalities is crucial for optimizing patient outcomes. Moreover, recent evidence suggests that targeted pulmonary hypertension therapies administered prior to pulmonary endarterectomy may optimize surgical outcomes. In fact, Castro et al.^8^ have demonstrated the potential benefit of incorporating these drugs into a multimodal treatment strategy. Future research should focus on larger, prospective studies to further evaluate the long-term benefits of Riociguat, as well as its integration with surgical and interventional approaches such as PEA and BPA, to optimize the treatment of CTEPH patients.

A key limitation of this study is its retrospective design and small sample size, which may limit the generalizability of the findings. As a retrospective observational study, the results are subject to selection and information bias, as well as unmeasured confounding factors. Notably, a significant change was not identified in Pulmonary Vascular Resistance (PVR) following treatment. The lack of significant hemodynamic improvement may reflect limited sample size, variability in treatment duration, or timing of catheterization, which may not reflect peak therapeutic response. This is consistent with prior real-world observations in similar populations.^3^

Furthermore, the lack of a control group precludes direct comparisons with other therapeutic approaches. However, the study's strength lies in being the first Brazilian cohort to provide detailed pre- and post-treatment data, including hemodynamic parameters, thereby contributing valuable real-world evidence on Riociguat use in Brazil.^9^^,^^10^ Importantly, access to Riociguat remains challenging in Brazil, with significant barriers related to medication approval, funding, and healthcare system navigation. This highlights the urgent need for policy interventions and expanded availability. Enhancing access to this therapy is essential, as real-world evidence demonstrates that Riociguat contributes to meaningful clinical improvements in exercise capacity, symptom burden, and biomarker levels. Delayed or limited access may prevent eligible patients from achieving these outcomes, reinforcing the importance of health policy strategies that prioritize equitable access to approved treatments for CTEPH patients in Brazil. Future efforts should focus on streamlining approval processes, securing funding mechanisms, and increasing physician and patient awareness about therapeutic options. To improve access to Riociguat in Brazil, advocacy efforts for its inclusion in public formularies and broader coverage by the national public health system should be prioritized.

## Conclusion

This study reinforces the effectiveness of Riociguat in treating CTEPH in a real-world Brazilian cohort, demonstrating meaningful improvements in functional status, biomarkers, and risk stratification. While hemodynamic changes were not statistically significant, the overall clinical response aligns with findings from international studies. These results highlight the therapeutic potential of Riociguat and underscore the urgency of expanding access to this treatment in Brazil.

## Data transparency

De-identified datasets used in this study are available upon reasonable request from the corresponding author.

## Authors’ contributions

CJCSF conceived the study. WSF, JLAJr, and CVPJ collected the data. TMV and MTF drafted the manuscript. CJCSF and RS critically reviewed the final version.

## Funding

None.

## Declaration of competing interest

The authors declare no conflicts of interest.
